# A MATE-Family Efflux Pump Rescues the *Escherichia coli* 8-Oxoguanine-Repair-Deficient Mutator Phenotype and Protects Against H_2_O_2_ Killing

**DOI:** 10.1371/journal.pgen.1000931

**Published:** 2010-05-06

**Authors:** Javier R. Guelfo, Alexandro Rodríguez-Rojas, Ivan Matic, Jesús Blázquez

**Affiliations:** 1 Departamento de Biotecnología Microbiana, Centro Nacional de Biotecnología-Consejo Superior de Investigaciones Científicas (CSIC), Madrid, Spain; 2 INSERM, 1001, Faculté de Médicine, Université Paris Descartes, Paris, France; Baylor College of Medicine, United States of America

## Abstract

Hypermutation may accelerate bacterial evolution in the short-term. In the long-term, however, hypermutators (cells with an increased rate of mutation) can be expected to be at a disadvantage due to the accumulation of deleterious mutations. Therefore, in theory, hypermutators are doomed to extinction unless they compensate the elevated mutational burden (deleterious load). Different mechanisms capable of restoring a low mutation rate to hypermutators have been proposed. By choosing an 8-oxoguanine-repair-deficient (GO-deficient) *Escherichia coli* strain as a hypermutator model, we investigated the existence of genes able to rescue the hypermutable phenotype by multicopy suppression. Using an *in vivo*-generated mini-MudII4042 genomic library and a mutator screen, we obtained chromosomal fragments that decrease the rate of mutation in a *mutT*-deficient strain. Analysis of a selected clone showed that the expression of NorM is responsible for the decreased mutation rate in 8-oxoguanine-repair-deficient (*mutT*, *mutY*, and *mutM mutY*) strains. NorM is a member of the multidrug and toxin extrusion (MATE) family of efflux pumps whose role in *E. coli* cell physiology remains unknown. Our results indicate that NorM may act as a GO-system backup decreasing AT to CG and GC to TA transversions. In addition, the ability of NorM to reduce the level of intracellular reactive oxygen species (ROS) in a GO-deficient strain and protect the cell from oxidative stress, including protein carbonylation, suggests that it can extrude specific molecules—byproducts of bacterial metabolism—that oxidize the guanine present in both DNA and nucleotide pools. Altogether, our results indicate that NorM protects the cell from specific ROS when the GO system cannot cope with the damage.

## Author Summary

Some bacteria and eukaryotic cells produce a higher-than-normal number of mutations (so-called “mutators”). Because some of the mutations produced can be favorable (such as antibiotic resistance in bacteria or resistance to anticancer drugs in human tumor cells), the high mutation rate may provide a short-term advantage. However, the production of huge numbers of mutations may compromise the future of these cells because they also accumulate disadvantageous mutations. Consequently, cells may contain backup mechanisms to reduce the accumulation of mutations. We have found that some types of hypermutable mutants can escape this fate by increasing the expression of an efflux pump predicted to export specific oxidative substances, the precursors of many mutations, and consequently reducing their number. Amazingly, this over-expression may confer several advantageous phenotypes simultaneously, such as antibiotic resistance, protection against reactive oxygen species and antimutability.

## Introduction

Maintaining the integrity of genetic information is crucial for all living organisms. Mutations originate from replication errors and DNA damage from endogenous and exogenous origin. Evolution, through natural selection, has produced a number of systems to prevent or repair these errors. The post-replication mismatch repair system (MMR) repairs mainly replication errors (for a review see [1]). Endogenous damage to DNA bases are repaired primarily by base excision repair (BER) (for a review see [2]). Of particular importance are oxidative DNA lesions which play a major role in spontaneous mutagenesis, because oxidized bases can mispair with noncognate ones [2]. Especially noteworthy amid these oxidative lesions is oxidation of guanine to 7,8-dihydro-8-oxo-2′-deoxyguanosine (8-oxo-dG). Guanine is particularly susceptible to oxidation on account of its low redox potential. If 8-oxo-dG is not repaired, it can be bypassed by DNA polymerases and pair with either C or A, causing GC to TA transversions [2].

Because 8-oxo-dGTP is highly mutagenic, efficient sanitizing mechanisms have evolved in all living cells to mitigate its highly mutagenic potential. In *E. coli* there are at least three proteins, MutM, MutY and MutT, engaged in avoiding the mutagenicity of 8-oxo-dGTP. These specialized proteins are known as the GO system [3]. MutM removes 8-oxoG paired with C in DNA, while the MutY protein removes A opposite 8-oxoG resulting from A mispaired with unrepaired 8-oxoG during replication. *E. coli* mutants defective in MutM or MutY exhibit higher than wild-type GC to TA spontaneous transversions [3]–[5]. The MutT enzyme is a nucleoside triphosphate pyrophosphohydrolase that converts 8-oxo-dGTP to 8-oxo-dGMP and pyrophosphate, thereby inactivating this mutagenic activity. In the absence of MutT there is an increase in AT to CG mutations [3].

Despite the high mutational burden produced by the absence of MMR or GO systems, naturally-occurring hypermutable *E. coli* and *Pseudomonas aeruginosa* isolates deficient in these systems have been found [6]–[10]. Hypermutable *E. coli* strains (mutators) can increase in frequency in laboratory bacterial populations under specific conditions that select rapid adaptation to environmental changes [11]–[13]. However, cells with the mutator phenotype pay a high biological price of deleterious mutations in the long run, which may result in extinction [14], [15]. Theoretical and experimental evidence of this cost has been obtained in bacterial populations propagated in the laboratory when submitted to severe bottlenecks [16]–[18].

Several possible ways to reduce the mutation rates of mutator strains can be envisaged, among these, mutations in additional loci that are able to reduce the mutation rate have been obtained in laboratory populations of *mutT* mutators submitted to long-term evolution [19]. However, after more than 20 years, the molecular mechanisms responsible for these compensatory phenotypes have not yet been described. Thus, our initial hypothesis raised the question of whether some back-up mechanisms may have evolved to alleviate the high cost of hypermutability in the absence of the original antimutator function.

In this work, we looked for genes that, when over-expressed, reversed or reduced the *mutT* mutator phenotype. To this end, we constructed and explored an *in vivo* library of *E. coli* genomic DNA fragments using a mini-Mu transposable element and the Mu phage [20]. We found that multicopy expression of *norM* is able to decrease the mutation rate in GO-repair deficient strains. NorM is the prototype of the multidrug and toxin-extrusion (MATE) family of cation-coupled efflux pumps, which includes many bacterial and eukaryotic members [21].

## Results

### Search for multicopy suppressors of the mutator phenotype of a *mutT* strain

**Figure 1:**
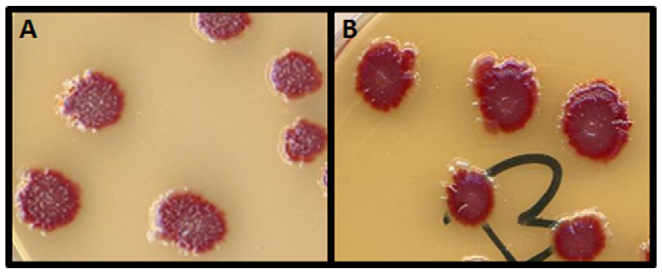
The papillation assay. The strain GLF1 Δ*mutT*::Kan Mu *cts* produces red colonies (Ara^−^) on arabinose-tetrazolium chloride agar plates. Ara^+^ revertants are spontaneously produced by mutation and appear as white microcolonies growing out of the surface of the main red colonies. The Ara^−^→Ara^+^ reversion rate can be visualized by the number of white papillae appearing per colony on tetrazolium-arabinose plates incubated for 7 days. A: the mutator strain GLF1 Δ*mutT*::Kan Mu *cts* forms colonies with a high number of Ara^+^ papillae (white); B: the strain GLF1 Δ*mutT*::Kan Mu *cts* harboring the mini-Mu plasmid with the chromosomal fragment containing *norM* forms colonies with a low number of papillae.

**Figure 2:**
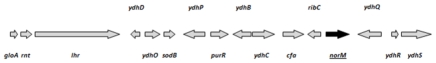
ORFs (arrows) present in the isolated chromosomal fragment.

As stated in the Materials and Methods section, the mini-Mu system produces a plasmid overexpression library of chromosomal fragments by homologous recombination between two adjacent mini-Mu transposons. We screened for GO^−^ strains with a reduced mutation rate using the colony papillation screen described in figure 1. The screen of the mini-Mu library (about 1,500 clones) yielded several colonies with a clearly reduced number of white Ara^+^ papillae on agar plates containing arabinose and tetrazolium chloride. One of them, showing a papillation pattern similar to the wild-type strain was chosen for further study (Figure 1). Plasmid DNA was purified, retransformed into the original Δ*mutT* strain and retested with the Ara^−^→Ara^+^ reversion assay. The ends of the fragment contained in the mini-Mu plasmid were sequenced and the chromosomal region between them inferred. This region included 16 genes, 8 with assigned functions (*gloA*, *rnt*, *lhr*, *sodB*, *purR*, *cfa*, *ribC* and *norM*) (Figure 2). In principle, we considered *sodB*, which encodes the Fe-dependent superoxide dismutase, and *norM*, which encodes an orthologue of the MATE family [21]–[23], as major candidates responsible for the decreased mutation rate in the *mutT*-deficient background. Overexpression of *sodB* may reduce the level of reactive oxygen species (ROS) in the cell, leading to a reduced level of 8-oxo-dG, which consequently compensates for the absence of MutT activity. On the other hand, NorM is the prototype of the MATE family of cation-coupled transporters, which characteristically possess 12 putative transmembrane domains and have been reported in all three kingdoms of life [21]. Expression of NorM conferred resistance to several agents, such as norfloxacin, aminoglycosides and ethidium bromide, *via* a mechanism requiring the proton motive force [24]. Interestingly, MATE proteins have been described as exporters of toxic organic cations and guanidine [25], rendering NorM an excellent candidate for the export of oxidative precursor molecules.

### *norM* is the antimutator gene

Plasmids containing the genes *sodB* [pCsodB] and *norM* [pCnorM] were obtained from the Complete Set of *E. coli* K-12 Open Reading Frame Archive (ASKA) library [26]. These plasmids, harboring the two cited genes cloned into the pCA24N vector, were transformed into the host strain GLF1 Δ*mutT*::*kan* [F′CC101] (Table 1), a strain that assays AT to CG Lac^+^ mutations. Several transformants carrying either pCsodB or pCnorM, and the parental vector pCA24N, were analyzed by a Lac^+^ reversion papillation assay. A clear decrease in the number of Lac^+^ papillae was observed in the strain harboring pCnorM, but not in those harboring pCsodB or the vector pCA24N (data not shown).

**Table 1:** Bacterial strains, plasmids and phages used in this study.

*S*trains, plasmids and phages	Relevant genotype; phenotype	Source or reference
**strains**		
MEC222 (scavenger)	MG1655 *lacZ*ΔT::*cat*; Lac^−^, Cm^R^	[49]
MC4100	*araD139* Δ (*argF lac*)*205 flbB5301 ptsF25 relA1 rpsL150 deoCI*	[53]
Pop3001.6	MC4100 *malT*::Mu *c*ts [pEG109]	Laboratory collection
BW25113	*rrnB3* Δ*lacZ4787 hsdR514* Δ(*araBAD*)*567* Δ(*rhaBAD*)*568 rph-1*	[46]
JW0097	BW25113 Δ*mutT*::Kan; Kan^R^	[46]
JW1648	BW25113 Δ*sodB*::Kan; Kan^R^	[46]
JW1655	BW25113 Δ*norM*::Kan; Kan^R^	[46]
NR10831	*ara*, *thi*, Δ*prolac* [F′ CC101]; Rif^R^, Nal^R^; assays AT to CG transversions	[45]
GLF0	NR10831 Δ*norM*::Kan; Kan^R^	This work
GLF1	NR10831 Δ*mutT*::Kan; Kan^R^	This work
GLF1 Mu *cts*	NR10831 Δ*mutT*::Kan Mu *cts*; Kan^R^	This work
GLF2	NR10831 Δ*mutT*	This work
GLF3	NR10831 Δ*norM*::Kan; Kan^R^	This work
GLF4	NR10831 Δ*mutT* Δ*norM*::Kan; Kan^R^	This work
GLF5	NR10831 Δ*mutT*::Kan; Kan^R^	This work
NR10834	*ara*, *thi*, Δ*prolac* [F′ CC104]; Rif^R^, Nal^R^; assays CG to TA transversions	[45]
GLF6	NR10834 Δ*mutM::bla*; Amp^R^	This work
GLF7	NR10834 *mutY::*Tn*10*; Tet^R^	This work
GLF8	NR10834 Δ*mutM::bla mutY::*Tn*10*; Amp^R^ Tet^R^	This work
MG1655	F^−^ λ^−^ *ilvG rfb*50 *rph*-1	Laboratory collection
GLF9	MG1655 Δ*mutT*	This work
GLF10	NR10831 Δ*sodB*::Kan; Kan^R^	This work
**phages**		
Mu *c*ts	Mu *c*ts62 (temperature-sensitive repressor)	[54]
MudII4042	Mu *c*ts62 A^+^ B^+^ *cat* (Cm^R^) rep_Pl5A_ *lac*('*ZYA*)931	[54]
**plasmids**		
pEG109	MudII4042::*phoA-proC*	[51]
pCP20	pSC101^ts^ *flp*	[55]
pCA24N	Ori ColE1 Cm^R^ *lacI* ^q^ *P_T5lac_histag::GFPuv4*	[26]
pCnorM	*norM* in pCA24N	[26]
pCsodB	*sodB* in pCA24N	[26]

Table 2 shows the mutation rates of strains NR10831, GLF0 (Δ*norM*::*kan*) and GLF1 (Δ*mutT*::*kan*) harboring plasmids pCA24N or pCnorM. No differences were observed between the wild-type and the mutant Δ*norM*::*kan*, and the presence of plasmid pCnorM did not modify mutation rates. However, the plasmid pCnorM produced a dramatic decrease in the mutation rate when introduced into the Δ*mutT* strain (Table 2). These results suggest that, in the absence of additional damage, other protective mechanisms may suffice to cope with this damage, and that the anti-mutator effect of NorM can be observed only when mutagenesis is increased by the absence of a key antimutator mechanism such as MutT.

**Table 2:** Spontaneous Lac^−^ to Lac^+^ mutation rates of the wild-type, Δ*mutT* and Δ*norM* derivatives containing the empty vector or the *norM*-encoding plasmid.

Strain	Mutation rate^a^ (mutations/cell/generation)	Fold Increase
NR10831 [pCA24N]	1.7×10^−9^±6.8×10^−10^	1
NR10831 [pCnorM]	2.2×10^−9^±2.2×10^−10^	1.3
GLF0 Δ*norM*::Kan [pCA24N]	2.3×10^−9^±3.8×10^−10^	1.3
GLF0 Δ*norM*::Kan [pCnorM]	2.0×10^−9^±2.3×10^−10^	1.2
GLF1 Δ*mutT*::Kan [pCA24N]	3.3×10^−7^±1.7×10^−8^	194
GLF1 Δ*mutT*::Kan [pCnorM]	4.8×10^−10^±2.8×10^−10^	0.3

a mean ± SEM of the mutation rates for 3 independent experiments are shown.

### Effect of *norM* on the Val-R mutation rate

The *norM* antimutator effect was observed for a *lac* marker on the F′*pro-lac* episome. This may indicate a phenomenon similar to the process of “stress-induced mutation”, which may occur preferentially in the F′ episome [27]. To test this possibility, we used acquisition of valine-resistance (Val-S to Val-R) to verify the antimutator properties of NorM for a chromosomal marker. The valine-resistance (Val-R) mutation assay has been used previously by others [28], [29]. Table 3 shows that plasmid pCnorM is able to reduce the frequency of Val-R mutants of the strain GLF9 Δ*mutT* by two orders of magnitude, as in the case of the *lac* reversion assay.

**Table 3:** Spontaneous Valine^R^ mutant frequencies.

Strain	Mutant Frequency^a^ (mutants/viable cell)	Fold Increase
MG1655 [pCA24N]	3.4×10^−7^±6.2×10^−8^	1
GLF9 Δ*mutT*::Kan [pCA24N]	5.2×10^−4^±6.6×10^−5^	1,530
GLF9 Δ*mutT*::Kan [pCnorM]	4.7×10^−6^±2.8×10^−6^	13.6

a mean (± SEM) of the mutant frequencies for 4 independent experiments are shown.

In conclusion, the experiments described above show that the expression of NorM in a multicopy plasmid reverses or reduces the mutator phenotype caused by the lack of MutT activity at both episomal and chromosomal markers.

### Effect of *norM* expression on the mutator phenotype of other GO-repair-deficient strains

The *norM* effect might be due to the active extrusion of toxic metabolites involved in the oxidative damage of guanine. In this case, the same effect, i.e., a decrease in mutation rate, should also be observed in the *mutM mutY* background, because both MutM and MutY remove errors caused by the presence of 8-oxodG in the DNA. This double mutant has an elevated rate of GC to TA transversions [3]. The frequency of Lac^+^ mutants was measured for strain NR10834 and its mutant derivatives GLF6 (*mutM*), GLF7 (*mutY*) and GLF8 (*mutM mutY*) containing either pCA24N or pCnorM (Table 4). All these strains carry the *lacZ* missense allele present in the episome F′CC104 [30], which reverts to Lac^+^ uniquely by a GC to TA transversion mutation. The *mutM* mutant shows a slight increase in mutant frequency, and both the *mutY* and the double *mutM mutY* mutants show a high mutant frequency (one and two orders of magnitude, respectively, in relation to the wild-type strain NR10834) (Table 4). The expression of *norM* from the multicopy plasmid decreased the mutant frequency of these three mutants significantly (*P* values<0.05 in all cases; see Table 4 for values). These mutant frequencies were even below that of the wild-type. At this stage, unfortunately, we have no satisfactory explanation for this phenomenon, although the transcriptional or postranscriptional regulation of the activity of other systems cannot be ruled out.

**Table 4:** Spontaneous Lac^−^ to Lac^+^ mutant frequencies of the wild-type and *mutM*, *mutY* and *mutM mutY* derivatives with or without the *norM*-encoding plasmid.

Strain	Mutant frequency^a^ (mutants/viable cell)	Fold Increase
NR10834 [pCA24N]	4.2×10^−8^±1.4×10^−8^	1
GLF6 Δ*mutY*::*bla* [pCA24N]	8.9×10^−7^±1.5×10^−7^	21.2
GLF6 Δ*mutY::bla* [pCnorM]	1.1×10^−9^±2.0×10^−10^	0.03
GLF7 *mutM*::Tn*10* [pCA24N]	1.4×10^−7^±1.3×10^−7^	3.3
GLF7 *mutM*::Tn*10* [pCnorM]	7.2×10^−10^±1.4×10^−10^	0.02
GLF8 *mutM mutY* [pCA24N]	6.1×10^−6^±1.0×10^−6^	145
GLF8 *mutM mutY* [pCnorM]	1.2×10^−9^±7.5×10^−10^	0.03

a mean (± SEM) of the mutant frequencies for 4 independent experiments are shown. *P*-values were obtained for pairwise comparisons by the nonparametric Mann–Whitney U test. The mutant frequency of the wild-type strain, NR10834 [pCA24N], is significantly different from those of the strains Δ*mutY*::*bla* [pCA24N] (*P* = 0.016), Δ*mutY::bla* [pCnorM] (*P* = 0.009), *mutM*::Tn*10* [pCnorM] (*P* = 0.014), *mutM mutY* [pCA24N] (*P* = 0.009) and *mutM mutY* [pCnorM] (*P* = 0.009).

These results strongly suggest that the *norM* effect is due to an active extrusion of toxic metabolites involved in the oxidative damage of guanine, even when 8-oxodG is incorporated into the DNA.

### Multicopy expression of *norM* protects against killing by hydrogen peroxide in a *mutT*-deficient background

**Figure 3:**
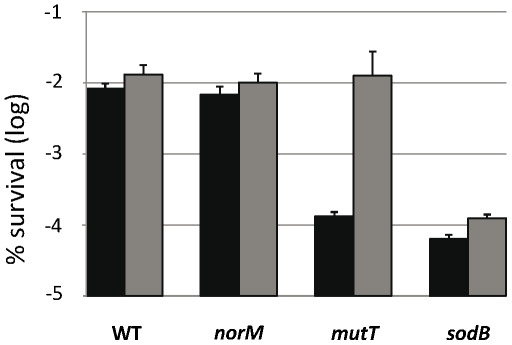
Viability after H_2_O_2_ treatment. The data represent survival percentages after 30 min of 50 mM H_2_O_2_ treatment. Data for strains NR10831, NR10831Δ*norM*, NR10831Δ*mutT*, and NR10831Δ*sodB* harboring either the empty vector pCA24N (black) or the plasmid expressing *norM*, pCNorM, (gray) are shown. Survival is represented as the percentage of cfu after H_2_O_2_ treatment relative to before treatment. The error bars indicate one standard error of the mean of four independent replicates.

GO-deficient cells are more sensitive to H_2_O_2_-induced killing than those of the wild-type [31], [32] *via* a mechanism that is still unknown. This has led us to investigate the effect of *norM* expression on cell protection against H_2_O_2_-induced killing. Figure 3 shows that *norM* expression in the multicopy plasmid promotes evident protection for *mutT* cells from H_2_O_2_-induced killing (*P* = 0.009). However, this protection is not statistically significant in the wild-type background (*P* = 0.6) (Figure 3). As in the case of mutagenesis, these results suggest that NorM protects cells from oxidative damage mainly in the absence of a key protective mechanism and when this damage is increased, e.g. in the presence of ROS-generating substances. Therefore, it is conceivable that NorM could protect cells from oxidative damage in the absence of a key cellular ROS-protective mechanism, such as superoxide dismutases [33]. When we tested the effect of *norM* expression in a *sodB* mutant background (lacking the Fe-dependent form of superoxide dismutase), we found that *norM* expression promotes only a minor protection, statistically not significant (*P* = 0.12), against H_2_O_2_ in this sensitive background (Figure 3).

Altogether these results show that NorM offers evident protection against H_2_O_2_-killing when the GO system cannot cope with the damage, although it provides no protection in the absence of other ROS protective mechanisms, such as superoxide dismutase SodB, or in the wild-type background. This suggests that NorM acts almost exclusively as a backup of the GO system, and is capable of alleviating both the increased oxidative DNA damage as well as the mutagenesis produced by the lack of this key system.

### Effect of *norM* expression on intracellular ROS levels

**Figure 4:**
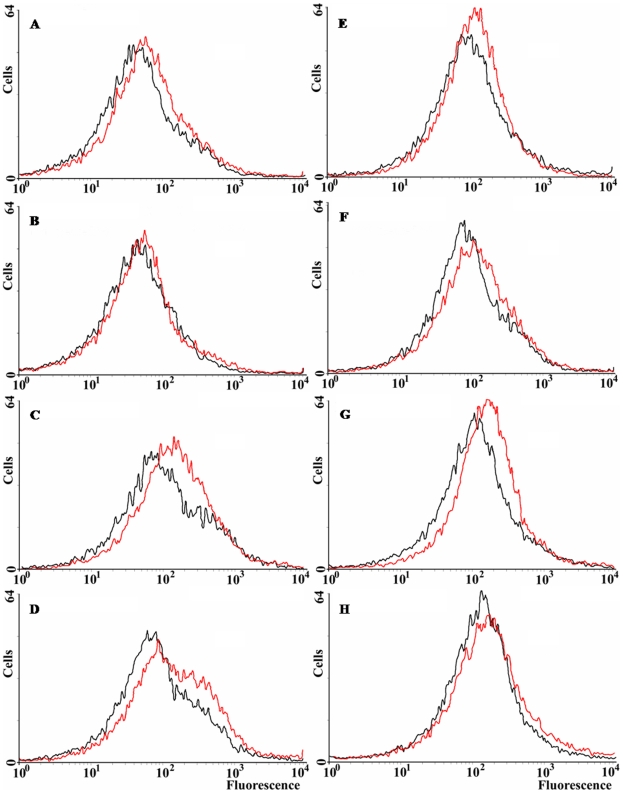
ROS levels in *E. coli* wild-type and *norM*, *mutT* and *sodB* derivatives. Representative histograms plotting the spontaneous fluorescence of 15,000 non-treated (A–D) and H_2_O_2_-treated (E–H) cells, revealed by DHR, as measured by flow cytometry. Cells containing either the empty vector or the *norM*-containing plasmid are represented as red or black lines, respectively. A and E: wild-type strain (non treated and H_2_O_2_-treated, respectively); B and F: *norM*-deficient strain; C and G: *mutT*-deficient strain; D and H: *sodB*-deficient strain.

All the previous results suggest that *norM* expression can reduce the intracellular ROS levels under specific circumstances. We have examined ROS levels qualitatively *via* the use of dihydrorhodamine 123 (DHR) and flow cytometry. DHR is a probe for the detection of intracellular reactive oxygen species. It is oxidized into rhodamine 123, which produces a maximal emission at 529 nm when excited at 507 nm (Enzo Life Sciences). Figure 4 shows that *norM* expression in the multicopy plasmid pCNorM can reduce the amount of reactive oxygen species slightly, but consistently, in the wild-type strain (Figure 4A). However, the same plasmid produced a greater reduction in the ROS intracellular level of the Δ*mutT* strain compared to the wild-type (Figure 4C) (note that the ROS intracellular levels, measured as oxidized rhodamine 123, are represented in a logarithmic scale). Expression of *norM* reduces the ROS level only slightly in the *sodB*-defective background (Figure 4D). In parallel experiments, ROS intracellular levels were measured after H_2_O_2_ treatment for 30 min. As expected, treated cells produced higher levels of ROS in all cases (the histograms are shifted slightly to the right), with minor reductions produced by the expression of *norM*, compared to values from cells harboring the empty vector (Figure 4E–G). The higher effect was observed in the *mutT*-deficient background (Figure. 4G). Once again, and in agreement with the data from the H_2_O_2_-protection experiments, our results suggest that the expression of *norM* acts almost exclusively as a back-up mechanism of the GO-system.

### Effect of *norM* expression on protein carbonylation

**Figure 5:**
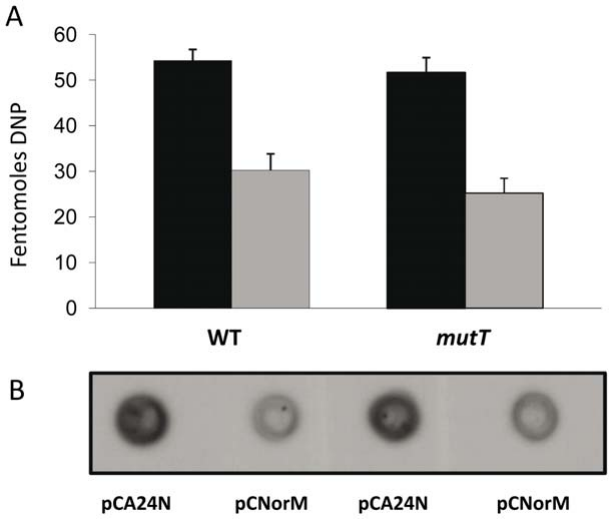
Protein carbonylation. Carbonylation is observed in the wild-type and *mutT*-derivative strains containing either the empty vector or the plasmid expressing *norM*, following treatment with 10mM H_2_O_2_ for 15 min. A: Bar graph quantifying the protein carbonylation (femtomoles of DNP) in cells containing the empty vector pCA24N (black bars) or the *norM*-plasmid pCNorM (gray bars) in the wild-type (left bars) and *mutT* strains (right bars). The data are the mean values from four separate experiments and error bars represent one standard error. B: Representative blot showing the accumulation of protein carbonyl groups in H_2_O_2_ challenged cells.

One of the most important effects of an increased intracellular ROS level is protein carbonylation. Thus, we studied the effect of *norM* expression on protein carbonylation in H_2_O_2_-treated and non-treated wild-type and Δ*mutT* cells. The level of spontaneous protein carbonylation in both the wild-type and *mutT* non-treated cells growing in the exponential phase was undetectable with the OxyBlot kit. Nevertheless, when submitted to H_2_O_2_ pre-treatment, the expression of *norM* in the multicopy plasmid pCNorM produced a consistent decrease in the amount of carbonylated proteins in both the wild-type NR10831 and the Δ*mutT* strains (Figure 5A and 5B).

## Discussion

The integrity of genetic information is a critical process for life. Consequently, a number of systems have evolved to prevent or repair replicative and postreplicative errors. The GO system, which is able to prevent and repair oxidative damage produced by the oxidation of deoxy-guanine to 8-oxo-dG, is a key component of the antimutation cell machinery. However, despite the great mutational burden produced by the absence of the GO system, naturally-occurring hypermutable *E. coli* and *P. aeruginosa* isolates deficient in *mutT* have been found [6], [10].

Hypermutable *E. coli* strains (mutators) show a selective advantage over the wild-type as they can produce more favorable mutations. In fact, it has been demonstrated that mutators can increase in frequency under specific conditions of environmental change in laboratory bacterial populations [11]–[13].

However, hypermutation may represent a colossal evolutionary cost for bacteria because most of the mutations are neutral or deleterious [14], [15]. Theoretical and experimental evidence of this cost has been obtained in laboratory propagated bacterial populations when they were submitted to severe bottlenecks [16]–[18]. The accumulation of deleterious mutations in genes experiencing relaxed selection or no selection at all will cause a more rapid fitness loss if mutators later encounter environments in which those genes are important [34], [35]. Thus, from a theoretical stand-point, hypermutator populations must reduce the mutation rate or face the possibility of extinction, at least under specific conditions. There are three possible ways to reduce the mutation rate for a mutator: (i) reverse the mutation that produced the mutator allele; (ii) replace the mutator allele with a wild-type gene from a non-mutator cell *via* horizontal gene transfer; and (iii) compensatory mutations at additional loci. Two studies strongly suggest that the replacement of the mutator allele with a non-mutator by horizontal transfer may have occurred in nature [36], [37]. Evidence of the third way, i.e., mutations at additional loci that reduce the mutation rate, has been obtained in laboratory populations of mutators submitted to long-term evolution, although the genes responsible for this have not been characterized [19]. Moreover, some antimutator mutations in the α catalytic subunit of the DNA-polymerase III compensate the high mutation rate of *mutT*-deficient strains and *dnaQ* (proofreading-deficient) mutators, such as *mutD5*, by increasing replication fidelity [38]–[40]. Finally, a different mode is to reduce the cost of accumulating deleterious mutations (deleterious load). Increased levels of the heat-shock chaperones, DnaK and GroEL, in lineages that accumulate many mutations, reduce the fitness cost produced by deleterious load [41].

The hypothesis we tested was that some back-up mechanisms could alleviate the high cost of hypermutability in the absence of the original antimutator function. We chose deficiency in the GO-repair system as a model. Our strategy was to look for genes which, when over-expressed, rescue or reduce the *mutT* mutator phenotype. Our results indicate that the expression of a pump from the multidrug and toxin extrusion (MATE) family is able to counteract the mutagenic effect of the lack of *mutT*, completely restoring the basal levels of AT to CG transversions. Interestingly, Yang et al [42] found that the overproduction of *emrR*, encoding a negative regulator of the multidrug resistance pump EmrAB, led to a mutator phenotype, suggesting a link between multidrug efflux and mutagenesis.

NorM is the prototype of the MATE family of cation-coupled transporters, which include many bacterial members [21]. Curiously, one of these members is *E. coli* DinF, which also seems to be involved in the efflux of DNA damage-inducing compounds (Guelfo, J.R. et al, unpublished data). Based on sequence similarity, two human MATE transporter genes, hMATE1 and hMATE2, have been described [43]. hMATE1 is primarily expressed in the liver, skeletal muscle and the kidney, whereas hMATE2 is expressed in testis. It is thought that mammalian MATE transporters mediate the final step in the excretion of toxic organic cations [43]. The existence of these NorM orthologues in mammalian cells suggests that they might play a similar antimutator role, protecting the cells from endogenous and exogenous mutagenic metabolites.

Several lines of evidence strongly suggest that NorM extrudes molecules that oxidize dG to 8-oxo-dG, when present in both the DNA and the dNTP pool: (i) overexpression of *norM* is also able to reduce the elevated rate of GC to TA transversions induced by the lack of both MutM and MutY activities, which act on errors produced when 8-oxo-dG is present in the DNA; (ii) there is a decrease of intracellular ROS and carbonylated protein levels in the *mutT* background; and (iii) there is significant protection of *mutT*-deficient cells from H_2_O_2_-induced killing.

The fact that overexpression of NorM reduces mutagenesis in MutM MutY-deficient cells (which do not have increased ROS levels but rather decreased removal of 8-oxo-dG from DNA), but not in the wild-type cells, is intriguing. An alternative hypothesis is that in wild-type cells most spontaneous mutagenesis is not caused by 8-oxo-dG, but is rather due to other problems and pathways. Therefore, lowering ROS levels in the wild-type cells does not affect those pathways or spontaneous mutant frequencies. However, in MutM MutY-deficient cells, the mutagenesis caused by 8-oxo-dG exceeds the levels from the normally main spontaneous mutation pathways. Once this happens, either because cells are *mutM mutY* or because they were treated with H_2_O_2_, GO-mediated mutations dominate and these are reduced by NorM.

Altogether our results suggest that NorM may act as a specific backup mechanism able to alleviate both oxidative DNA damage and mutagenesis when the GO system is impaired. The nature of the oxidizing molecules putatively extruded by NorM remains unknown. However, the apparent substrate specificity of NorM provides a good starting point to understanding why GO-deficient cells are more sensitive to H_2_O_2_-induced killing than those of the wild-type.

Concerning the evolutionary aspect, the major goal of this work is to demonstrate that a mutator phenotype can be reverted by extragenic-based mutation. To our knowledge, this kind of mutator-phenotype rescue has not been described before and may provide an explanation as to how some naturally-occurring hypermutator populations can avoid losing fitness by deleterious load. This striking discovery suggests that the surprisingly high proportion of *E. coli* mutators deficient in a repair pathway (up to 1%) in nature [6]–[8] could have been even higher, because some of the mutator phenotypes may have been hidden by extragenic “compensation”. In any case, irrespective of possible overproduction of NorM in nature, this overproduction has been necessary to discover the antimutator and ROS-protective effect of NorM.

The over-expression of *norM* in bacteria has been associated with multiple drug resistance [25], [44]. Here we reveal that this over-expression may confer several advantageous phenotypes simultaneously, such as antibiotic resistance, protection against ROS and antimutability.

## Materials and Methods

### Strains and constructions

The strains, phages and plasmids used in this study are listed in Table 1. The strains with the F′*pro-lac* episomes NR10831 [F′CC101] and NR10834 [F′CC104] carry the *lacZ* marker on these F′ episomes containing specific mutations that can revert to Lac^+^ by only one defined mutational event [30], [45]. These strains were kindly provided by Dr. I. Fijalkowska (Institute of Biochemistry and Biophysics, Warsaw, Poland). Strains NR10831Δ*norM*::Kan (GLF0), NR10831Δ*mutT*::Kan (GLF1) and NR10831Δ*sodB*::Kan (GLF10) were constructed by P1 transduction of the alleles from strains BW25113Δ*norM*::Kan (JW1655), BW25113Δ*mutT*::Kan (JW0097) and BW25113Δ*sodB*::Kan (JW1648), respectively, obtained from the Keio collection, NARA Institute (http://ecoli.aist-nara.ac.jp) [46]. Strains NR10834 Δ*mutM*::*bla* (Amp^R^) and NR10834 *mutY*::miniTn*10* (Tet^R^) were constructed by P1 transduction from strains BW853Δ*fpg*::*bla* and CSH11 *mutY*::miniTn*10* [47], respectively.

The vector used in this study was pCA24N, harboring a ColE1 replicon and a predicted copy number per cell of around 20 [26] (Table1). The plasmids pCnorM and pCsodB contain the wild-type *norM* and *sodB* genes, respectively, cloned in the vector pCA24N. These genes are transcribed from the pT5/*lac* promoter, which is repressed by the product of the *lacI* gene. Despite the predicted strict repression, our data with GFP fusions indicate that most genes cloned in this plasmid are transcribed, even in the absence of IPTG (data not shown). Plasmids were obtained from the Complete Set of *E. coli* K-12 Open Reading Frame Archive (ASKA) library [26].

### Media

All strains were grown routinely in Luria-Bertani (LB) [48]. The arabinose papillation assay was carried out in tetrazolium arabinose, which contains (per litre) tryptone (10 g), yeast extract (1 g), NaCl (5 g), agar (16 g), arabinose (10 g) and tetrazolium chloride (0.05 g). Lactose reversion and valine-resistance assays were conducted in M9 minimal medium, as described below. For M9 minimal medium (MM) preparation, M9 salts (Sigma) were supplemented with thiamine (2.5 mg/l), MgSO4 (1 mM) and amino acids (0.04 mg/ml), when required. Glucose or lactose were used as carbon source at 2 mg/ml final concentration. Solid and soft medium contained 15 g/l and 7 g/l of agar, respectively.

Valine resistance assays were performed in glucose MM and the plates were supplemented with valine (0.04 mg/ml). Lac reversion assays were carried out in MM. Inocula were grown with glucose as carbon source and the plates were supplemented with lactose as unique carbon source according to Miller [48]. The scavenger strain MEC222 [49], harboring a truncated *lacZ* allele (*lacZ*ΔT::*cat*) with the C-terminal region replaced by the *cat* cassette (Table 1), was added to the lactose agar MM before being spread (40 µl of a stationary-phase culture per litre of media, approximately 10^7^ cells/plate). Plates were stored overnight at room temperature. The desired cultures for Lac reversion assays were spread in a M9 top-agar layer, without carbon source and supplemented with 5-bromo-4-chloro-3-indolyl-3-D-galactoside (X-Gal), as described by Miller [48].

When required, antibiotics were added to the media: kanamycin (Km) 50 µg/ml, chloramphenicol (Cm) 40 µg/ml, ampicillin (Amp) 100 µg/ml and tetracycline (Tet) 20 µg/ml.

### DNA manipulations and phages handling

DNA manipulations have been described previously [50]. Plasmid DNA was routinely extracted by alkaline lysis and transformed into *E. coli* strains by the CaCl_2_ method [50]. Procedures for handling bacteriophages Mu and P1 have been described [48].

### Antimutator screen

MudII4042 was used to construct an *in vivo* random library of *E. coli* chromosomal fragments into a multicopy plasmid [51]. MudII4042 is a derivative of the Mu bacteriophage that contains the P15A replication origin and the chloramphenicol-resistance gene. This mini-Mu element can transpose at high frequency when de-repressed and it can be replicated in a lytic growth when present with a helper Mu *c*ts phage. The heat-induced lysate of a MudII4042 Mu *c*ts strain, Pop3001.6 in this case, produces a variety of packaged DNA. Sequences flanked by two copies of this mini-Mu can be packaged along with them. After infection, homologous recombination can occur between the mini-Mu sequences, resulting in the formation of plasmids carrying the transduced sequences. This library was transduced into the GLF1 (Δ*mutT*) strain (Table 1) and plated onto arabinose-tetrazolium agar plates with chloramphenicol. The hypermutable Δ*mutT* strain produces red colonies with a high number of white papillae as a result of the spontaneous reversion of Ara^−^ to Ara^+^. The plates were incubated for a total of 7 days and examined at daily intervals for colonies with decreased reversion to Ara^+^, as visualized by the number of white papillae appearing per colony. About 1,500 clones were analyzed for the Ara^−^→Ara^+^ reversion rate. The clones with an evident decrease in this rate (low number of Ara^+^ papillae) were selected, their plasmids purified, retransformed into the original Δ*mutT* strain, retested and preserved for further analysis. The plasmid from one of them, presenting a papillation pattern similar to the wild-type (*mutT* ^+^) strain, was sequenced and analyzed in detail. The sequences at the ends of the cloned fragments were analyzed using BLAST searches, thereby identifying the region cloned in the mini-Mu-derived plasmid. This plasmid contained several genes, some of which were considered by us as better candidates to reduce mutation rate. To find the gene responsible for this decreased papillation we used the appropriate plasmids, pCsodB and pCnorM, from the Complete Set of *E. coli* K-12 Open Reading Frame Archive (ASKA) library. This library contains each *E. coli* open reading frame cloned into the pCA24N vector [26]. Plasmids harboring the candidate genes were taken from this collection and transformed into the host strain GLF1 (Table 1). All transformants were analyzed by Lac^+^ reversion frequency.

### Mutation rate and mutant frequency measurements

To calculate mutation rates pre-inocula were initiated in tubes with 3 ml of M9 glucose directly from frozen samples. The pre-inocula were grown at 37°C overnight to stationary phase. From each culture less than 10^5^ cells were inoculated in 100 ml of M9 glucose and divided into 10 independent cultures, 10 ml each and less than 10^4^ cells/culture. These inocula were grown for 24 hours. Appropriate dilutions of the saturated cultures were plated on selective media valine-MM or Lac-X-gal-MM to determine the number of valine resistant mutants or Lac^+^ mutants, respectively. LB plates were used to determine the total colony-forming units (cfu). Mutation rates (number of mutants per cell per division) were estimated by the method described [52]. To calculate mutant frequencies (number of mutants per total cell count), the mean number of mutants per millilitre was determined and divided by the average number of cfu per ml. Experiments were repeated at least three times.

### Flow cytometry

Flow cytometry analysis was performed using the H_2_O_2_-activated fluorescent dye Dihydrorhodamine 123 (DHR) (Enzo Life Sciences). Wild-type and mutant derivatives were grown in M9 at 37°C, as described above, and then each one was split into two cultures (one control and one treated with 50 mM H_2_O_2_) and incubated for 30 min. Cells (0.5 ml/culture) were pelleted by centrifugation, resuspended in saline containing 15 µM DHR, and then incubated for 15 min and diluted 1:50 in phosphate-buffered saline. The fluorescence levels (excitation 488 nm and emission 530 nm) of 15,000 cells were then counted for each strain under each condition using a FACSCalibur cytometer (BD Biosciences). WinMDI (The Scripps Institute, Purdue University, USA) was used for data analysis and generation of histograms.

### Determination of the cellular level of protein carbonylation

Wild-type and mutant derivatives were grown, as described above, and then each was split into two cultures (one control and one treated with 50 mM H_2_O_2_) and incubated for 30 min. After the time indicated, peroxide was removed by centrifugation. Subsequently, the cells were washed and resuspended in M9 medium preheated to 37°C and further incubated. Cells were lysed as follows: 1 ml of the culture was washed with 50 mM Tris buffer (pH 7.5) and centrifuged for 10 min at 14,000 rpm. The pellet was re-suspended in 150 µl lysis buffer containing 0.5 mg/ml lysosyme, 20 µg/ml DNAse, 50 µg/ml RNAse, 1 mM EDTA, and 10 mM Tris (pH 8). 15µl of 10% SDS solution was added and the cells were incubated at 100°C for 5 min. In order to examine the level of protein carbonylation in these lysates, we used the Chemicon OxyBlot kit (Chemicon) to derivatize the carbonyl groups in the protein side chains to 2,4-dinitrophenylhydrazone (DNP-hydrazone) by reaction with 2,4-dinitrophenylhydrazine. These DNP derivative crude protein extracts were dot blotted onto a nitrocellulose membrane, which was incubated with primary antibody specific to the DNP moiety of the proteins, and subsequently incubated with secondary (goat anti-rabbit) horseradish peroxidase-antibody conjugate directed against the primary antibody. Carbonylation was observed by the ECL, enhanced chemiluminiscence, reagent (Amersham Pharmacia Biotec). The intensity of each dot was quantified by densitometry analysis using the Image Master VPS-CL software. The intensity of each dot was normalized to equal levels of protein, which were determined using Bradford reagent (Bio-Rad) and expressed in femtomoles of DNP, according to the control of the OxyBlot kit.

### Estimation of H_2_O_2_-induced cell death

The strains were grown at 37°C in M9 supplemented with appropriate antibiotics to mid-exponential phase and washed with 0.9% NaCl. The cells were treated with 50 mM H_2_O_2_ for 30 min at 37°C and washed with 1 ml of 0.9% NaCl. A non-treated control was also included. Appropriate dilutions were immediately plated onto LB plates and incubated overnight at 37°C to determine viability. Experiments consisted of five independent cultures for each strain. Cell survival was calculated by comparing the number of cfus of treated cells to those of the cells not treated.

### Statistical analysis

The statistical signification for pairwise comparisons was estimated by the Mann-Whitney U test. *P* values≤0.05 were considered to be statistically significant.
